# Alarmingly large unemployment gap despite of above-average education in adults with ASD without intellectual disability in Germany: a cross-sectional study

**DOI:** 10.1007/s00406-022-01424-6

**Published:** 2022-05-14

**Authors:** Julia Espelöer, Julia Proft, Christine M. Falter-Wagner, Kai Vogeley

**Affiliations:** 1 Department of Psychiatry, University Hospital Cologne, Kerpener Strasse 62, 50924 Cologne, Germany; 2 Department of Psychiatry, Medical Faculty, LMU Munich, Nussbaumstrasse 7, 80336 Munich, Germany

**Keywords:** Autism, Employment, Education, General German population, Adults

## Abstract

For individuals with autism spectrum disorder (ASD), both getting access to as well as staying in the labor market are very challenging. However, the detailed educational, vocational, and employment characteristics of persons with ASD without intellectual disabilities are not yet studied. We conducted a retrospective study on a sample of 232 clinically late-diagnosed adults with ASD without intellectual disabilities. Data were compared to the general German population obtained from the public database of the German Federal Employment Agency. Results showed that the majority of persons with ASD graduated from high school and obtained a university entrance qualification (ASD: 50.4%; general population: 32.5%). Also, lower rates of basic secondary education were found in the ASD sample (ASD: 16.5%, general population: 29.6%). Significantly less individuals with ASD completed vocational training (40.1%) in comparison to the German population (56.3%). Despite the above-average level of education, the unemployment rate of the sample substantially exceeds that of the general population by the factor 5 (ASD: 25.2%; general population: 5.2%). Periods of unwanted unemployment of persons with ASD lasted on average 23 months with interpersonal problems being the main reason for contract termination. A higher level of educational qualification does not protect against a higher risk of unemployment for individuals with ASD presumably due to autism-specific interpersonal difficulties. Data emphasize the necessity to develop and spread both specific employment support activities for individuals with ASD as well as adequate awareness raising strategies. Funded by a public grant of the “Landschaftsverband Rheinland (LVR)”.

## Introduction

One of the most important milestones in the transition from adolescence to adulthood is entering working life. An unsuccessful transition can have far-reaching consequences for further socialization and may lead to social exclusion [1, 2]. Yet even if this transition is accomplished, working environments with their complex social dynamics pose further challenges, especially for individuals with autism spectrum disorder (ASD) for whom deficits in social interaction skills and stereotyped, repetitive behavior are characteristic [1, 3–5]. Qualitative impairments in processing social information intuitively may add to the risk of exclusion. Individuals with ASD describe requirements imposed by their workplace with a ratio of 80% social to 20% working skills, which is a misfit to their own strengths and weaknesses [6]. Indeed, communicative difficulties, inappropriate social behaviors and adherence to ritualistic and routine behavior in adulthood are reported to hinder access to and maintenance of employment [4–9]. Fragmented professional career paths due to an increased number of job terminations interspersed with long periods of unemployment can be the consequence [4, 6, 8]. When reviewing the literature on concrete employment rates in ASD, previous research indicates unemployment rates between 39 and 73% in the USA [10, 11] and 24 to 54% in Great Britain [5, 7, 12], whereby no differentiation was made with regard to the severity of autism symptoms or the level of functioning and age at diagnosis. Further research suggests that the majority of early-diagnosed adults with ASD with and without intellectual disabilities are employed in sheltered or supported settings [5, 13]. Especially, the psychosocial outcome of young adults with ASD lags behind that of adults with other impairments and comparable demographic and disability characteristics [1–3, 12, 14]. Many adults with ASD remain dependent on their parental home or other institutional sources of support [5]. Individuals with ASD are at heightened risk of poorer post-secondary educational and vocational outcomes when showing greater functional impairments [4]. Taylor and Seltzer [13] indicated that young adults with ASD and co-occurring intellectual disability were more likely to have post-high school employment compared to individuals with ASD without intellectual disabilities. However, especially late-diagnosed adults with ASD without intellectual disabilities appear to be neglected in professional support services compared to individuals with other disabilities such as learning or language disabilities and other mental health problems [2, 7, 12, 13, 15]. Despite high education levels, where normal employment rates could be expected, individuals with ASD suffer from high rates of unemployment thus demonstrating a need for specific support systems [11, 15–18].

Research on unemployment rates in Germany suggests similar findings as obtained in international studies of unemployment rates in the range of 13.5 and 58% [18–20]. For the first time, the German general population was included in the current study as a control group and was quantitatively compared with occupational qualifications and unemployment rates in a large clinical sample of late-diagnosed individuals with ASD without intellectual disabilities. The current study corroborates findings of Maslahati and colleagues [21] of an imbalance of increased educational levels as well as increased unemployment rates in adults with ASD with and without intellectual disabilities compared to the general population of Germany. The occupational status of individuals attending the adult autism outpatient clinic was assessed at the beginning of the diagnostic process; all individuals were without experiences of ASD-specific professional support service. Similar previous studies either solely studied the influence of special interests on occupational success [18] or on the classification of occupation [20]. The comparison of an ASD sample with the general German population by Maslahati [21] was extended by quantitative approximation between groups. The survey period of our study—2014–2019—follows previous research (2009–2011 by Riedel et al. [19]; 2009–2014 by Frank et al. [20]; sampling period was not provided by Kirchner and Dziobek [18]). The purpose of the current study was to provide detailed education and employment characteristics by focusing on the specific group of late-diagnosed high-functioning adults with ASD in comparison with the general population in Germany.

## Method

### Participants

Retrospective data were obtained from the Adult Autism Outpatient Clinic, Department of Psychiatry, of the University Hospital of Cologne, Germany. Registration for diagnostics in the outpatient clinic requires a referral and thus a prior assessment by a specialist. 3964 individuals registered for diagnostic clarification during the period of 2014–2019 received a questionnaire [22] before their first interview to assess their occupational status of which 1765 questionnaires were returned corresponding to a response rate of 45%. 443 individuals were clinically diagnosed with ASD according to ICD-10 criteria of which 232 individuals provided a completed questionnaire [23]. IQ testing is not standard in the diagnostic process. Due to educational qualification level in our sample, the exclusion of cognitive disabilities in the ASD sample can be assumed [24]. The average age of the ASD sample was 35 years (19–67), 67 individuals (28.9%) were female, 165 (71.1%) were male. The catchment area of the sample consists of 43.5% of individuals who came from the wider area of Cologne, the rest of the sample came from other regions in Germany. The manuscript was submitted to the local Ethics Committee of the Medical Faculty, University of Cologne, which confirmed that the study is exempt from the requirement of ethical approval as under German law no separate ethics application to and statement of ethical approval by the local ethics committee are required for performing purely retrospective clinical studies.

### Instruments

The vocational questionnaire consists of two parts [22]. The first part, which is reported in the current analysis, captures descriptive data about formal education level, occupational skill level, employment status, psychosocial situation, periods of unemployment, and frequency and reasons of terminations (For the German translation of levels of formal qualification see Table 1). The second part is composed of the two subscales ‘workplace experiences’ and ‘wishes and requirements of an ideal workplace’. Ratings in the second part were performed using five-point Likert scales. Data of the ASD sample was compared to whole population data obtained from the public databases of the German Federal Employment and the German National Education Report [25–27]. These databases are not updated yearly so that we selected the 2018 data from the aforementioned institutions that were closest to our data collection period.

**Table 1:** Levels of formal qualification

School education, summarized	School education	German		Required School attendance
University entrance qualification	General university entrance-level qualification	“Allgemeine Hochschulreife”	Upper secondary level	12 or 13 years
University entrance qualification	Discipline-specific university entrance-level qualification	“Fachhochschulreife”	Upper secondary level	12 or 13 years
Other qualifications	General certificate of secondary education	”Realschulabschluss”	Lower secondary level	10 years
Other qualifications	Basic secondary education^a^	“Hauptschulabschluss”	Lower secondary level	9 years

Levels of formal qualifications with related German translation

^a^Compulsory education lasts 9 or 10 years, depending on the federal state of the Federal Republic of Germany

## Results

### Education and vocational qualification

Results for the successful general school education at least reaching basic secondary education were comparable between groups, *Χ*^2^(1) = 1.1, *p* = 0.29. More specifically, results show that in the ASD group, significantly more individuals reached the highest possible school education of a general or discipline-specific university entrance-level qualification (*Χ*^2^(1) = 33.7, *p* < 0.001), complementary to a significantly lower proportion of individuals with ASD who only reached a basic secondary education, as compared to the general population (*Χ*^2^(1) = 18.9, *p* < 0.001) (see Table 2).

**Table 2:** Highest qualification achieved by group

Education	Group			
	ASD	Population	*Χ*^*2*^_(1)_	*p*
University entrance-level qualification	50.4	32.5	33.7	< .001*
General certificate of secondary education	27.0	29.9	0.9	.330
Basic secondary education	16.5	29.6	18.9	< .001*
No school-leaving certificate/unspecified^a^	6.1	7.8	0.9	.333
Vocational Qualification				
Vocational Qualification	61.7	74.2	19.1	< .001*
Academic degree	21.6	17.9	2.1	.147
Vocational training	40.1	56.3	24.8	< .001*
In process of graduation	10.8	8.9	1.0	.316
Sheltered training measure	4.3	n.p		
Without completed vocational qualification	16.8	16.5	0.02	.899

Values in % by group

*Group*: *ASD *autism spectrum disorder (2014–2019), *Population *German general population (2018), *Χ*^2^ Chi-square goodness of fits test

*p < .05

^a^It is possible that some participants are still in education at the time of the survey

After school-based education, significantly less individuals with ASD obtained vocational qualification compared to the general population (*Χ*^2^(1) = 19.1, *p* < 0.001). Significant differences were found for vocational training, with significantly less completed qualifications in the ASD group compared to the general population (*Χ*^2^(1) = 24.8, *p* < 0.001). In the ASD group, increased rates of academic degrees were found, but differences were not significant (see Table 2).

### Employment

**Fig. 1:**
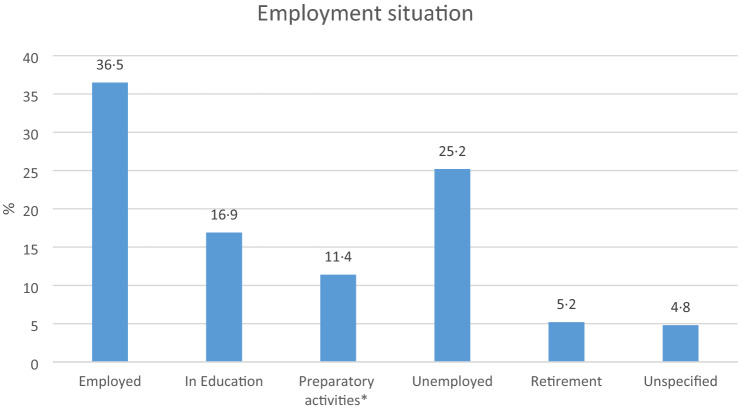
Employment situation of the ASD group in % (not available for the general German population). *Preparatory activities = Supportive interventions such as sheltered workplace or rehabilitation, side job, internship

36.5% of the participants in the ASD group reported to be employed (full-time, part-time, self-employed, civil servant). 16.9% were in education (school, vocational training, studies) and 11.4% of participants underwent preparatory activities (supportive interventions, side jobs, internships), 25.2% of individuals with ASD were unemployed, 5.2% were already retired (Fig. 1).

The majority of the ASD sample with a university entrance qualification were employed (44.3%). One-fifth were affected by unemployment (21.7%) or were in education (21.7%), respectively. Moreover, one-third of all graduates with a secondary education was employed (31.3%) or unemployed (32.3%) (Table 3).

**Table 3:** Employment status by school education and vocational qualification of the ASD group

	Employment status in %						
School Education	Employed	In education	Preparatory activities	Unemployed	Retirement	Unspecified	Total
University entrance qualification	44.3	21.7	6.1	21.7	2.6	3.5	100
Secondary education	31.3	11.1	11.1	32.3	7.1	7.1	100
No school-leaving certificate	7.1	14.3	57.1	7.1	14.3	0.0	100
Average	36.4	16.7	11.4	25.4	5.3	4.8	100
Vocational Qualification							
Academic degree	65.3	8.2	4.1	14.3	6.1	2.0	100
Vocational training	45.2	6.5	7.5	30.1	5.4	5.4	100
Without qualification	11.4	33.0	19.3	26.1	4.5	5.7	100
Average	36.5	17.0	11.3	25.2	5.2	4.8	100

Employment status by school education and vocational qualification of the ASD group in %

*Group*: *ASD *autism spectrum disorder (comparable detailed data not available for the general German population)

### Unemployment

Regarding the unemployment status, the ASD sample significantly exceeded the unemployment rate of the general population with 25.2% of ASD participants currently unemployed as compared to 5.2% in the general population, *Χ*^2^(1) = 187, *p* < 0.001 (not in Table). 98.3% of all unemployed individuals with ASD had a successful school education (general population: 82.4%), *Χ*^2^(1) = 10.1, *p* = 0.001, whereby 43.1% had completed a university entrance qualification (general population: 17.8%) (*Χ*^2^(1) = 25.4, *p* < 0.001) (Table 4).

**Table 4:** Level of highest school education achieved by unemployed individuals per group

Education	Group			
	ASD	Population	*Χ*^2^(1)	*p*
Successful school education	98.3	82.4	10.1	.001
University entrance qualification	43.1	17.8	25.4	< .001*
General university entrance-level qualification	25.9	11.6	11.5	< .001*
Discipline-specific entrance-level qualification	17.2	6.2	12.2	< .001*
General certificate of secondary education	31.0	21.5	3.1	.077
Basic secondary education	24.1	33.5	2.3	.131
No school-leaving certificate	1.7	17.6	10.1	.001*
Unspecified	–	9.7		

Level of school education reached by unemployed individuals by group in %

*Group*: *ASD *autism spectrum disorder (2014–2019), *Population *German general population (2018), *Χ*^2^ Chi-square goodness of fits test

^*^p < .05

When comparing the highest school degree achieved by unemployed persons, results show that significantly more unemployed individuals in the ASD group show general (*Χ*^2^(1) = 11.5, *p* < 0.001) or discipline-specific (*Χ*^2^(1) = 12.2, *p* < 0.001) university entrance-level qualifications compared to the general population, whereas the proportion of persons with ASD who did not reach a school-leaving certificate was significantly lower (*Χ*^2^(1) = 10.1, *p* = 0.001) (Table 4). Regarding the highest vocational qualification achieved, results did not significantly differ for unemployed individuals with academic degree and vocational training in both groups. The rate of unemployed persons who did not finish any vocational qualifications was increased in the general population compared to the ASD group, *Χ*^2^(1) = 4.4, *p* = 0.04 (Table 5).

**Table 5:** Level of highest vocational qualification achieved by unemployed individuals per group

Vocational Qualification	Group			
	ASD	Population	*Χ*^*2*^_(1)_	*p*
Academic education	12.3	7.9	1.5	.220
Vocational training	49.1	39.6	2.2	.142
Without completed vocational qualification/unspecified	38.6	52.4	4.4	.037*

Level of vocational qualification reached by unemployed individuals by group in %

*Group*: *ASD *autism spectrum disorder (2014–2019), *Population *German general population (2018), *Χ*^2^ Chi-square goodness of fits test

^*^p < .05

Participants reported periods of unemployment on average of 23 months, accordingly about two years on average (comparable detailed data not available for the general German population). 79.2% of all unemployed individuals with ASD were significantly more often long-term unemployed for at least 12 months (general population: 34.8%), *Χ*^2^(1) = 41.6, *p* < 0.001 [26].

### Rates and reasons of termination

The results showed an average termination frequency of persons with ASD of almost two terminations per person with a maximum up to twelve terminations. Interpersonal problems were cited significantly more often than professional problems as a reason for termination (*z* = 4323, *p* < 0.001). There were no differences between the rate of terminations given by the employer or by the employee (*z* = 2366, *p* = 0.47).

## Discussion

The current study corroborates the general pattern of a striking imbalance of the high level of education of persons with ASD without intellectual disability and their comparably low employment rate in Germany. For the first time, we are able to provide a quantitative approximation of a fivefold higher unemployment rate compared to the general population in Germany. Interpersonal difficulties were stated significantly more often than other reasons leading to termination in the ASD group.

Our study confirms and extends previous research by highlighting that a significantly higher level of education in the ASD sample, e.g. 50.4% university entrance-level qualifications (general population: 32.5%) and 21.6% graduate university (general population: 17.9%), does not protect from higher risk of unemployment. Previous studies in Germany with samples of late-diagnosed adults with ASD reported comparable results [19–21]. However, these studies examined smaller samples of individuals diagnosed with ASD according to ICD-10. In contrast to our results, in the general population the unemployment rate decreases the higher the school-leaving qualification. Contrasting to our sample of individuals with ASD, unemployed individuals in the general population are characterized by a relatively low qualification level (basic secondary education degree: 33.5%), and low rates (< 50%) of completed vocational training.

In the current study, individuals with ASD show significantly better school-leaving qualifications, including lower rates of basic secondary education in addition to higher rates of general university entrance-level qualifications (see Table 2). Furthermore, there is a higher rate of unemployed individuals with ASD with completed school education (see Table 4) as well as completed vocational qualifications (see Table 5) compared to the general German population. However, alarmingly high unemployment rates in the current sample of 25.2%, which exceed the unemployment rates in the German population of 5.2% by a factor of five, clearly demonstrate the challenges of stable integration as well as adaption to the demands of the labor market. It can be assumed that requirements for social skills, flexible behavior, quick adaptation to new processes as well as executive, structuring and planning skills increase in university and vocational training settings compared to lower level educational environments [28].

In particular, the context of vocational training and later workplace situations in terms of job interviews and practical requirements could add to the challenge for individuals with ASD. Due to the limited abilities of individuals with ASD in social skills and flexible behavior, coping with these requirements represent a major challenge. In the current study, significantly less individuals with ASD completed vocational training (40.1%) in comparison to the German population (56.3%). Our results were comparable to those in a previous German study by Frank and colleagues [20] with 43.1% who also included late-diagnosed adults with ASD without intellectual disabilities. Maslahati and colleagues [21] reported lower values (33%), which might be due to the broader sample of individuals with and without intellectual disabilities. Impairments in social skills as well as inflexible routines and ritualistic behavior might hinder the transition to successful employment with increased expectations for social skills, especially in adulthood [1, 9, 22].

Thus, the transition from school to the working life is a specific problem for persons with ASD that requires adequate measures like early intervention programs [29]. Early interventions need to be installed additionally for the first 2 years after leaving school, targeting the person-environment fit, the role of parents as well as a comprehensive, integrated support system [3, 12, 30].

Until today, there is clearly a lack of suitable support services for individuals with ASD without intellectual disabilities [3]. Parents are forced to be continuously supportive and engaged, because support ended after leaving school [7]. Reduced soft skills and adaptation to social rules as well as stereotypical behavior appear to be a barrier in the labor market and might result in repeated terminations. In the current sample, periods of unemployment on average of 23 months were reported, which are comparable results to previous research with 24.4 months [20]. Compared to the general German population (34.8%), individuals with ASD were significantly more often long-term unemployed (79.2%). In accordance with previous findings, interpersonal difficulties were reported to be the main reason for terminations in our sample [6]. 35% of the ASD sample report to have experienced job termination at least once due to interpersonal reasons and 63% of the participants reported to struggle due to high expectations of interpersonal relationships. Unfortunately, comparison data on terminations of employments are not available for the general population in Germany.

As a hypothesis for future research, we propose that especially adults with ASD without intellectual disabilities are at risk of being overlooked in supported employment services. Company-integrated workplaces are suggested to avoid segregated settings and thus social exclusion [31].

## Limitations

The present study examined individuals attending the Adult Autism Outpatient Clinic of the University Hospital of Cologne likely due to self-perceived or externally perceived difficulties and with preceding professional assessment. We were studying a specific group of patients from an outpatient clinic by focusing on late-diagnosed high-functioning adults without intellectual disabilities. It needs to be emphasized that results could not be generalized to all individuals with autism. A large sample size as well as a sufficient response rate of 45% was achieved based on data from individuals who were motivated and capable to complete the full questionnaire. Help-seeking behavior might be reduced in a sample of high-functioning autistic individuals, which should have tended to reduce the unemployment rate. Data from the general German population are not as fine-grained as the data from the ASD sample (e.g. missing information on terminations of employments). Although the ASD group represents a separately studied sample, it is still part of the general population, which was used for comparison. Comorbid disorders have been discussed as an important factor in research on the employment situation of people with ASD [1, 19]. This aspect has not been included in the current study and should be investigated as an important further aspect in future studies.

## Conclusions

The relation between educational achievements on one hand and employment rate on the other hand is clearly dysbalanced in persons with ASD compared to the general population. Professional supported employment programs are urgently needed aligning to long-term support for employees with ASD [22]. In addition, it is most important to increase societal knowledge and awareness about autism-related strengths and weaknesses addressing public authorities, potential employers, and colleagues. This should improve the hiring situation for adults with ASD and support stable employments.

## Data Availability

The datasets generated and analyzed in the current study are not publicly available as they are part of an ongoing project at the Adult Autism Outpatient Clinic, Department of Psychiatry, of the University Hospital of Cologne, Germany. The datasets are intended to be used for further analysis and publication, but are available upon request from the corresponding author.

## Abbreviations

ASD: Autism spectrum disorder
ICD-10: International Statistical Classification of Diseases and Related Health Problems 10th Revision
